# Transcriptome profiling analysis reveals that CXCL2 is involved in anlotinib resistance in human lung cancer cells

**DOI:** 10.1186/s12920-019-0482-y

**Published:** 2019-03-13

**Authors:** Jun Lu, Wei Xu, Jie Qian, Shuyuan Wang, Bo Zhang, Lele Zhang, Rong Qiao, Minjuan Hu, Yiming Zhao, Xiaodong Zhao, Baohui Han

**Affiliations:** 1 Department of Pulmonary Medicine, Shanghai Chest Hospital, Shanghai Jiao Tong University, 241 Huaihai West Rd, Shanghai, 200030 China; 2 Shanghai Center for Systems Biomedicine, Shanghai Jiao Tong University, 800 Dong Chuan Rd, Shanghai, 200240 China

**Keywords:** Anlotinib, Drug resistance, CXCL2, Non-Small Cell Lung Cancer, Transcriptome

## Abstract

**Background:**

Anlotinib has been demonstrated its anti-tumor efficacy on non-small cell lung cancer (NSCLC) in clinical trials at 3rd line. However, anlotinib resistance occurs during its administration, and the underlying mechanism is still unclear.

**Methods:**

Anlotinib resistant lung cancer cell line NCI-H1975 was established in vitro. Toxicologic effects undergoing anlotinib stress were observed upon NCI-H1975 cells and anlotinib resistant NCI-H1975 cells, respectively. Transcriptome profiling was performed to screen anlotinib resistance-associated genes between NCI-H1975 cells and anlotinib resistant NCI-H1975 cells. Functional assays were performed to examine the correlations between CXCL2 gene expression and anlotinib resistance.

**Results:**

We found anlotinib inhibits cell proliferation and cell viability in NCI-1975 cells, whereas it attenuates these activities in anlotinib resistant NCI-H1975 cells. Transcriptome profiling analysis identified 769 anlotinib-responsive genes enriched in the biological processes of microtubule-based process, cytoskeleton organization, and wound healing. Furthermore, we found 127 genes are associated with anlotinib resistance. In particular, we demonstrated that CXCL2 contributes to anlotinib resistance in NCI-H1975 cells.

**Conclusions:**

This study suggested that CXCL2 is involved in anlotinib resistance in NCI-H1975 cells and provided an insight for understanding the resistant mechanism of anlotinib.

**Electronic supplementary material:**

The online version of this article (10.1186/s12920-019-0482-y) contains supplementary material, which is available to authorized users.

## Introduction

Lung cancer is one of the most malignant cancers, and the non-small cell lung cancer (NSCLC) accounts for 80–85% of all lung cancer cases [1–3]. According to National Comprehensive Cancer Network (NCCN) guideline, the regimes of 1^st^ line and 2^nd^ line therapy bring great survival benefit for NSCLC patients, while the regimes of 3^rd^ line therapy have not been available so far [4, 5]. Anlotinib is an oral multi-targeted tyrosine kinase receptor inhibitor (TKI) [6]. Previous studies demonstrated that anlotinib inhibits proliferation and induces apoptosis of tumor cells via selectively inhibiting VEGFR (2/3), FGFR (1–4), and PDGFR (α/β) [7, 8]. Clinical trials have indicated anlotinib prolongs progression free survival (PFS) and overall survival (OS) of NSCLC patients significantly at 3^rd^ line [9–11]. However, the median PFS of 5.37 months suggests anlotinib resistance occurred at later stage.

Investigating the mechanisms of anti-tumor drug resistance will improve the therapeutic efficiency or promote novel agent discovery. Previous studies have showed the NSCLC patients harboring EGFR positive mutations (L858R or 19 Del) acquired drug resistance with median PFS of about 10 months when they were treated with the first generation TKIs (Gefitinib, Erlotinib and Icotinib) [12–15]. More studies revealed that T790 M mutation and other gene over-expression account for the majority [12, 16, 17]. Based on these findings, scientists have found the 3^rd^ generation TKI AZD9291 is effectively for the patients harboring T790 M mutation [18–20]. Investigating the resistant mechanism of anlotinib, a 3^rd^ line TKI, will be helpful to formulate the anlotinib-based therapeutic regime, and prolong PFS of NSCLC patients. In this study, we generated anlotinib resistant human lung cancer cell line NCI-H1975 in vitro, performed transcriptome analysis in NCI-H1975 cells and anlotinib resistant NCI-H1975 cells, and their anlotinib-treated counterpart with the aim to understand the mechanisms of anlotinib resistance.

## Materials and methods

### Cell culture

Human NSCLC cell line NCI-H1975 was purchased from ATCC (the global bioresource center, https://www.atcc.org/). TransDetect PCR Mycoplasma Detection Kit (TransGen, China) was used for excluding mycoplasma contamination. Cells were cultured in the mixed medium of 90% RPMI 1640 (Gibco, USA), 10% FBS (Gibco, USA), and 0.1 mg/ml streptomycin and 100 U/ml penicillin. All cells were incubated at 37°C in a humidified incubator containing 5% CO_2_.

### Generation of anlotinib resistant NCI-H1975

Anlotinib resistant NCI-H1975 was generated as previous reported [21, 22]. Briefly, 100 mg/ml ENU (sigma, USA) was exposed to totally 10^7^ NCI-H1975 cells for 24 h. And then, gradient concentration of anlotinib treatment was performed to induce anlotinib resistant NCI-H1975 cells. At first five days, NCI-H1975 cells were exposed to anlotinib (4 μg/ml) and the medium was changed every day. Then anlotinib (6 μg/ml, 8 μg/ml, 10 μg/ml and 12 μg/ml) treatments were performed in the next two months. The last cells (about 100 cells) showed viability when exposing to anlotinib (12 μg/ml). Lastly, after about one month’s proliferation, the anlotinib resistant NCI-H1975 cells were used to functional assays.

### Cell number and cell viability analysis

For evaluating the effects of anlotinib on cell growth, 10^5^ NCI-H1975 cells were seeded in each well of 6-well plates, and then exposed to anlotinib (8 μg/ml) for 24 h. Cell number changes in NCI-H1975 cells were observed with a phase-contrast microscope (Nikon, Japan). 10^3^ cells were seeded in 96-well plates and treated with anlotinib (8 μg/ml) for 24 h. Cell viability was determined using Cell Counting Kit 8 (CCK8, Dojindo, Japan) by spectrophotometric plate reader (Omega Bio-Tek, USA).

### RNA-seq library construction

RNA-seq library was performed as previous described [23–25]. Briefly, NCI-H1975 cells and anlotinib resistant NCI-H1975 cells were exposed to anlotinib (8 μg/ml) for 24 h. Total RNA was extracted by Trizol (Life Technologies, USA) according to the manufacturer’s protocol. mRNA was isolated from total RNA using Oligotex mRNA Mini Kit (Qiagen, Germany). Totally, 100 ng mRNA was used for library construction. After cDNA reverse-transcription, end repair and ligation, the original library was amplified 10–12 cycles in a thermal cycler using Q5 DNA Polymerase (NEB, USA). Lastly, the PCR products were performed by standard pair-end sequencing with 150 bp reads with Illumina Next500 (Illumina, USA). Raw data are available in the EMBL database under accession number E-MTAB-5997 and E-MTAB-7068.

### RNA-seq data processing

The sequencing quality of raw data was examined by FastQC software (Version 0.11.6). Qualified tags were mapped to reference genome (hg 38) by Tophat [26]. Cufflinks was used to characterize the differential transcription pattern [26]. Reads per kilo-base of transcript per million (RPKM) was used for detecting gene expression level. We performed differential gene expression analysis with RNA-seq data sets derived from NCI-H1975 and anlotinib-treated NCI-H1975, yielding 769 anlotinib-responsive genes. For the RNA-seq data sets generated with anlotinib resistant NCI-H1975 and anlotinib-treated anlotinib resistant NCI-H1975, we firstly removed genes whose transcription is modulated by anlotinib (RPKM in anlotinib resistant NCI-H1975 /RPKM in anlotinib-treated anlotinib resistant NCI-H1975 > 1), the resulting genes were intersected with anlotinib responsive genes and the cancer gene pool (http://www.bushmanlab.org/links/genelists), yielding 127 genes that are associated with Anlotinib resistance.

### Functional annotation and pathway analysis

According to our previous studies [23, 25, 27], functional annotation and pathway analysis were performed by public bioinformatics resource platforms named Database for Annotation, Visualization and Integrated Discovery (DAVID) and Panther Classification System. Briefly, a list of 769 anlotinib-responsive genes was uploaded to DAVID bioinformatics resources 6.7 and performed Gene Ontology (GO) analysis and Kyoto Encyclopedia of Genes and Genomes (KEGG) pathway [28], and a list of 127 genes was uploaded to PANTHER Classification System and performed Panther Pathway analysis [29].

### Wound healing scratch assay

10^5^ NCI-H1975 cells were seeded on 6-well plates for 24 h, and then were starved for 24 h. Scratch wound was performed using 200 μL pipet tip. Replace the medium with fresh 90% RPMI 1640 medium (Gibco, USA) and 10% FBS (Gibco, USA). Meanwhile, other wells were treated with anlotinib (4 μg/ml), CXCL2 (50 ng/ml, Pepro Tech, USA), alone or together. Phase-contrast microscope (Nikon, Japan) was performed to capture images. The migration rate was calculated based on the change of wound width.

### Cell invasion assay

Matrigel matrix (1:8 dilution, Corning, USA) was coated on transwell membrane. NCI-H1975 cells were seeded onto the top precoated chamber (5 × 10^4^ cells per well for 24 h evaluation; 2 × 10^4^ cells per well for 48 h evaluation) in 100 μL of FBS-free medium containing anlotinib (2 μg/ml) and CXCL2 (100 ng/ml, Pepro Tech, USA), alone or together. The medium containing 15% FBS (Gibco, USA) was placed in the bottom chamber. After 24 h or 48 h incubation, the invasive cells were fixed in 3.7% paraformaldehyde and stained with 0.1% crystal violet. Pictures were captured using fluorescence microscopy (Nikon, Japan). Cell numbers were examined by use of the microscopy affiliated software.

### Cell apoptosis assay

Cell apoptosis detection was performed as previous described [27]. Annexin V-FITC/PI apoptosis kit (Zoman Biotechnology Co., Ltd., China) was used to examine the cell apoptosis. NCI-H1975 cells were exposed to CXCL2 (100 ng/ml, Pepro Tech, USA) and anlotinib (4 μg/ml), alone or together for 24 h, and then stained with Annexin V-FITC and PI simultaneously. Flow cytometry (BD LSRFortessa, USA) was used for detecting apoptotic cells. PI-positive cells were designated end-stage apoptotic cells, and FITC-positive cells were designated early-stage apoptotic cells.

### Statistical analysis

There are at least three biological replicates, excluding RNA-seq, for each sample. GraphPad Prism 5 was use for histogram and statistical analysis. Student’s *t*-test was used to examine the raw data. Differences were considered significant at *P < 0.05, **P < 0.01 and ***P < 0.001.

## Results

### Anlotinib-induced cytotoxicity attenuates in anlotinib resistant NCI-H1975 cells

**Fig. 1:**
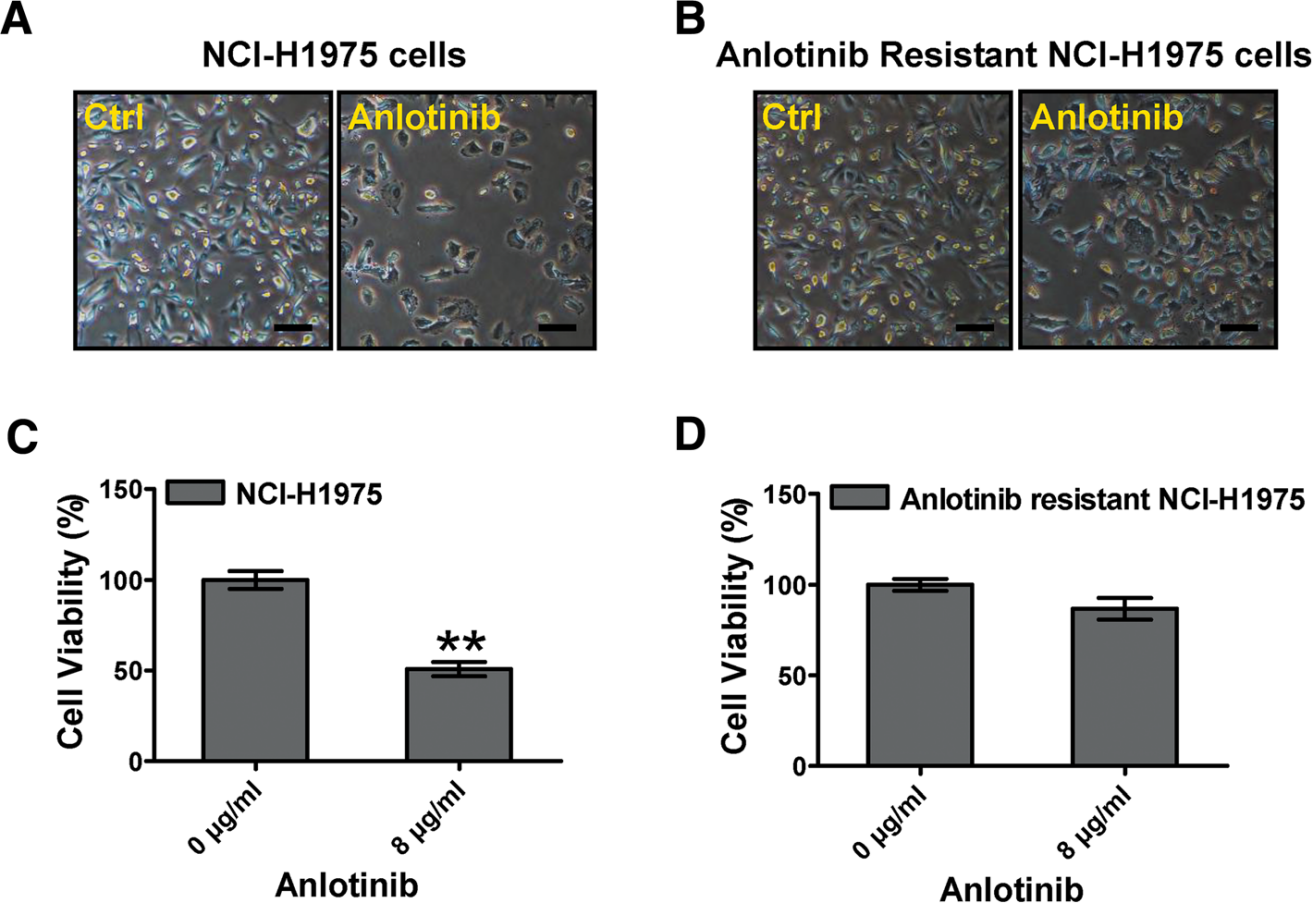
Effect of anlotinib on cytotoxicity on NCI-H1975 cells and anlotinib resistant NCI-H1975 cells. **a, b** NCI-H1975 cells and anlotinib resistant NCI-H1975 cells were treated with anlotinib (8 μg/ml) for 24 h, and then the cell numbers were captured by phase-contrast microscopy. Scale bar, 50 μm. **c, d** Cell viabilities were examined when NCI-H1975 cells and anlotinib resistant NCI-H1975 cells were exposed to anlotinib (8 μg/ml) for 24 h. Data are shown as the mean ± SD, *n* = 3, **P < 0.01

To understand the characteristics of anlotinib resistance, we firstly established anlotinib resistant NCI-H1975 cells in vitro. Under anlotinib stress, numbers of NCI-H1975 cells significantly decreased (Fig. 1a). However, numbers of anlotinib resistant NCI-H1975 cells were hardly affected, although exposing to same anlotinib stress as well (Fig. 1b). Furthermore, we examined the cell viability of these cells. After exposing to anlotinib for 24 h, the cell viability of NCI-H1975 was decreased remarkably. In contrast, the cell viability of anlotinib resistant NCI-H1975 was hardly effected (Fig. 1c, d). These results indicated that anlotinib-induced cytotoxicity attenuates in anlotinib resistant NCI-H1975 cells.

### Identification of anlotinib-responsive genes in NCI-H1975 cells

**Fig. 2:**
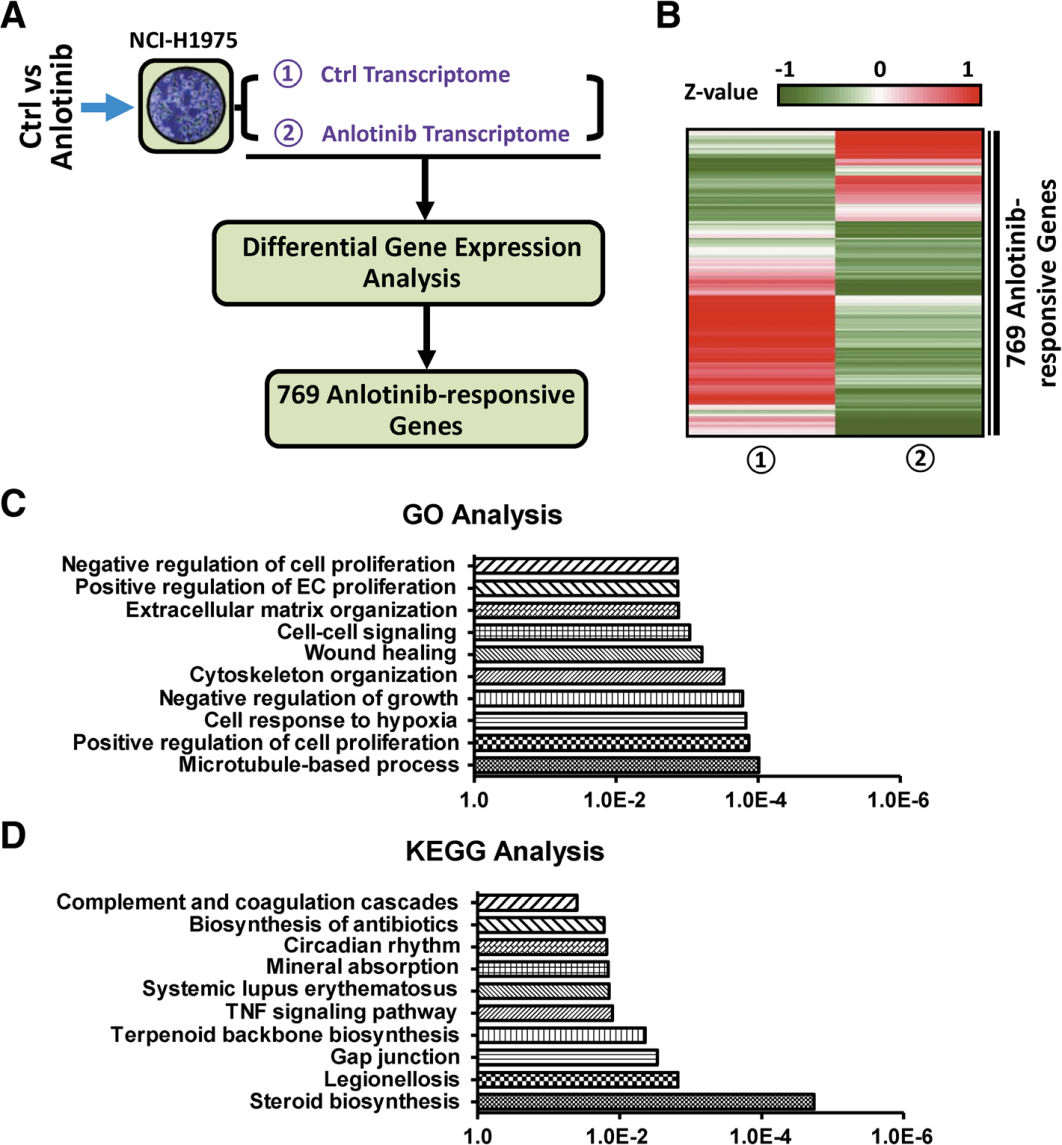
Identification of anlotinib-responsive genes in anlotinib-treated NCI-H1975 cells. **a** Flow diagram of the analysis for screening out 769 anlotinib-responsive genes. **b** Heat map representation of 769 anlotinib-responsive gene expressions upon NCI-H1975 cells and anlotinib-treated NCI-H1975 cells. **c** GO analysis of 769 anlotinib-responsive genes. **d** KEGG pathway analysis of 769 anlotinib-responsive genes

We reasoned that the expression of genes potentially involved in anlotinib resistance should be modulated upon anlotinib treatment. Thus, we firstly performed transcriptome profiling analysis by RNA-seq both in NCI-H1975 and anlotinib-treated NCI-1975 cells. We removed the genes marginally expressed in NCI-H1975 (RPKM < 1) and then carried out differential expression analysis (expression fold > 2). Compared with NCI-H1975, 769 genes were identified in anlotinib-treated NCI-H1975 (Fig. 2a, Additional file 1: Table S1). Heat map analysis suggested that 68% are down-regulated and 32% are up-regulated (Fig. 2b). Gene ontology (GO) analysis indicated that the 769 genes are significantly enriched in the biological processes of microtubule-based process, cytoskeleton organization, and wound healing (Fig. 2c). KEGG analysis suggested that these genes are enriched in the signaling pathways of steroid biosynthesis, gap junction, and TNF signaling pathway (Fig. 2d). Totally, these results suggest the set of 769 genes are anlotinib-responsive and involved in some cancer-related cellular activities.

### Identification of genes associated with anlotinib resistance in anlotinib resistant NCI-H1975 cells

**Fig. 3:**
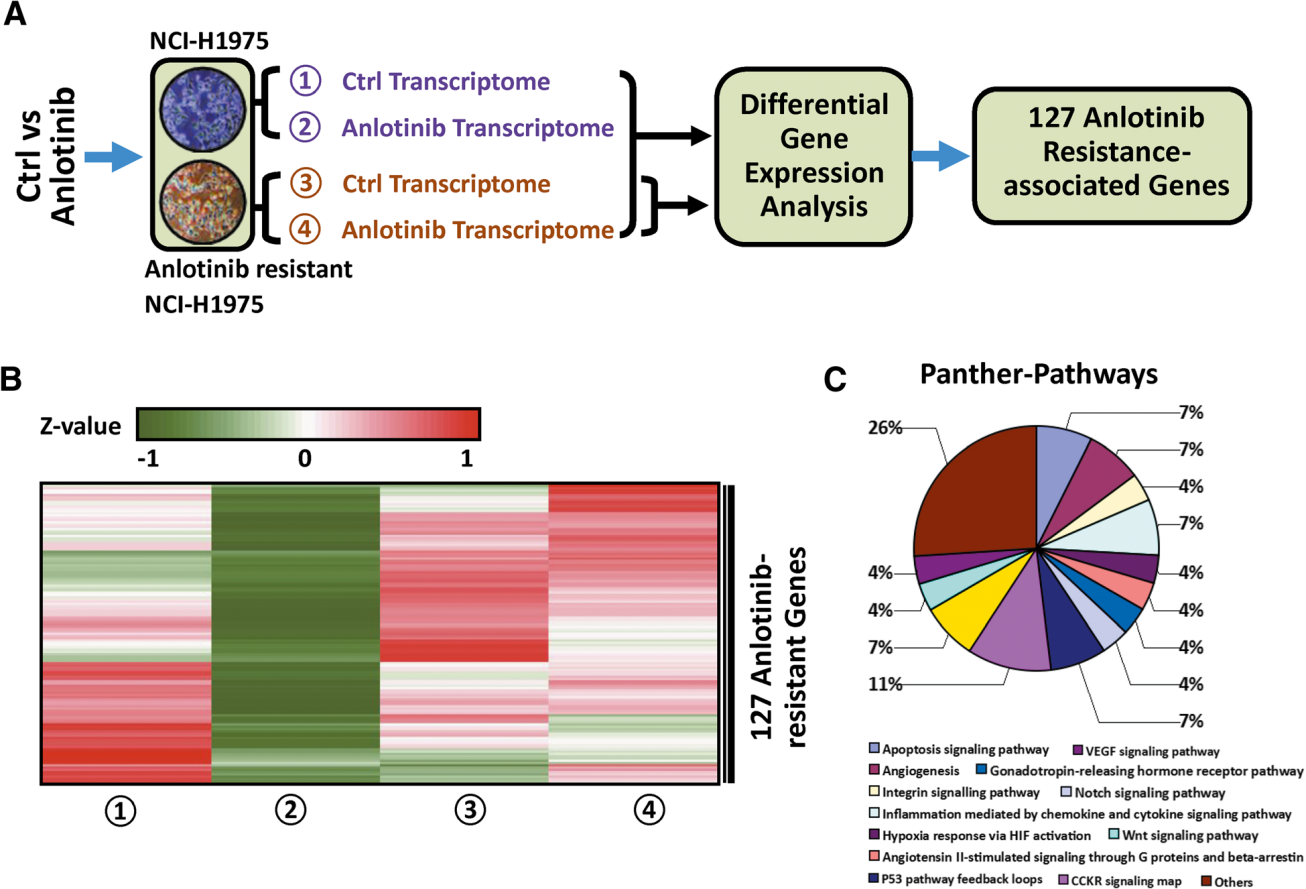
Identification of genes associated with anlotinib resistance in anlotinib resistant NCI-H1975 cells. **a** Flow diagram of multiple cross-check analysis for identifying 127 anlotinib resistance-associated genes. **b** Heat map representation of 127 anlotinib resistance-associated gene expressions in NCI-H1975 cells and anlotinib resistant NCI-H1975 cells, and their anlotinib-treated counterpart. **c** Panther-Pathway analysis of 127 anlotinib resistance-associated genes

To further understand the mechanisms of anlotinib resistance, we next performed RNA-seq analysis in anlotinib resistant NCI-H1975 cells and the anlotinib-treated counterpart. Among 769 anlotimib-responsive genes, we assumed the expression of genes that contribute to anlotinib resistance should be less modulated upon anlotinib treatment in anlotinib resistant cells (RPKM in anlotinib resistant NCI-H1975/ RPKM in anlotinib-treated anlotinib resistant NCI-H1975 < 1). This assumption allowed us to carry out differential gene expression analysis. Compared with anlotinib resistant NCI-H1975, 357 genes were identified in anlotinib-treated resistant NCI-H1975 cells with the criterion mentioned above. Furthermore, this set of 357 genes was compared with cancer gene pool (http://www.bushmanlab.org/links/genelists), yielding 127 genes (Fig. 3a, Additional file 2: Table S2). These genes are potentially involved in anlotinib resistance. As expected, the transcription levels of 127 genes decreased obviously in anlotinib-treated NCI-H1975 cells, while remaining less altered in anlotinib resistant NCI-H1975 and anlotinib-treated anlotinib resistant NCI-H1975 cells (Fig. 3b). Panther-Pathways analysis indicated that the 127 genes are involved in apoptosis signaling pathway, VEGF signaling pathway and angiogenesis (Fig. 3c).

### CXCL2 is involved in the resistance in anlotinib resistant NCI-H1975 cells

**Fig. 4:**
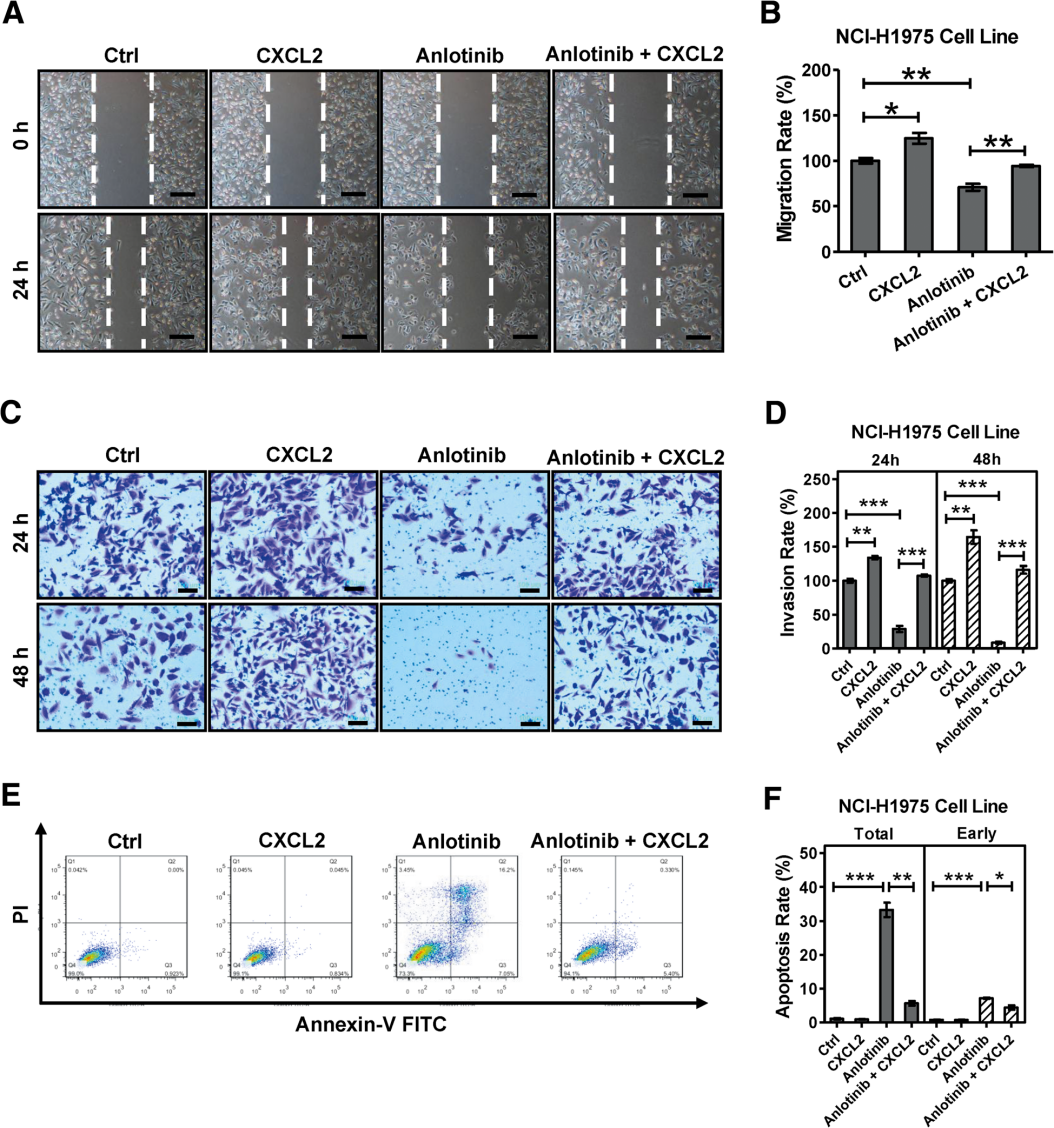
CXCL2 is involved in anlotinib resistance in NCI-H1975 cells. **a, b** CXCL2 (50 ng/ml) and anlotinib (4 μg/ml) were performed on NCI-H1975 cells, alone or together for 24 h. Migration rate was examined by wound healing scratch assay. Bars = mean ± SD, *n* = 3, *P < 0.05, **P < 0.01. Scale bar, 100 μm **c, d** CXCL2 (100 ng/ml) and anlotinib (2 μg/ml) were performed on NCI-H1975 cells, alone or together, for 24 h. Invasion rate was analyzed based on transwell assays. Bars = mean ± SD, *n* = 3, **P < 0.01, ***P < 0.001. Scale bar, 100 μm (E, F) NCI-H1975 cells were exposed to CXCL2 (100 ng/ml) and anlotinib (4 μg/ml), alone or together for 24 h. Ratio of total apoptosis and early apoptosis were examined based on flow cytometric detection. Data are shown as mean ± SD, *n* = 3, *P < 0.05, **P < 0.01, ***P < 0.001

Previous studies have demonstrated that CXCL2 plays an important role in anti-tumor drug resistance on various types of cancer, including breast cancer, colorectal cancer and glioblastoma [30–32], and modulates the property of migration, invasion and apoptosis in cancer cells [33, 34]. Interestingly, CXCL2 is one of 127 candidate genes potentially associated with anlotinib resistance. In this study, we observed some cellular migration-related GO items (cytoskeleton organization, microtubule-based process, and wound healing) and apoptosis signaling pathway are enriched when we investigated anlotinib resistance (Figs. 2c, 3c). We thus asked whether CXCL2 plays a role in the anlotinib resistance. To this end, we performed wound healing and transwell assays. We found exogenous CXCL2 obviously offsets anlotinib-induced NCI-H1975 cell migration inhibition (Fig. 4a, b). As shown in Fig. 4c and d, CXCL2 remarkably increases NCI-H1975 cell invasion, and promotes invasion of anlotinib-treated NCI-H1975 cells. Moreover, we found CXCL2 significant decreases anlotinib-induced apoptosis (both total apoptosis and early apoptosis) in NCI-H1975 cells (Fig. 4e, f). Overall, these results indicated CXCL2 is involved in the resistance in anlotinib resistant NCI-H1975 cells.

## Discussion

Our previous studies have shown that anlotinib is an effective anti-tumor agent for NSCLC at 3^rd^ line [9–11, 35]. It is also demonstrated that anlotinib selectively inhibits VEGFR (2/3), FGFR (1–4), and PDGFR (α/β) [7, 8]. However, drug resistance is inevitable in the last stage of cancer therapy.

NSCLC patients harboring EGFR (L858R and 19 Del) mutation received great benefit in more than 10 years ago, due to the finding of Gefitinib [36]. Although the first generation TKIs (Gefitinib, Erlotinib and Icotinib) have demonstrated its efficacy upon those NSCLC patients, acquired resistance will occurs after a median about 10 months therapy [12–15]. Subsequently, the scientists focused on the resistant mechanism study, and found that T790 M mutation, MET amplification, HER-2 mutation, and other gene alteration are contributed to acquired resistance [12, 16, 17]. Because of the patients harboring T790 M accounts for about 50% of the acquired resistance, scientists next screened out AZD9291, and brought greatly benefit for those patients with T790 M mutation [18–20].

In recent years, the high-throughput sequencing technology has served as an important platform to characterize the acquired resistance [37]. Understanding the underlying anlotinib resistant mechanism will benefit the therapeutic outcome. To address this issue, in the present study we performed transcriptome profiling analysis in NCI-H1975 cells, anlotinib resistant NCI-H1975 cells, and their anlotinib-treated cells. We identified a set of 127 genes that are potentially associated with anlotinib resistance via transcriptome profiling analysis (Fig. 3b).

Among these 127 candidate genes CXCL2 raised our attention. CXCL2 is an important cytokine, which usually is involved in wound healing, cancer metastasis, apoptosis, and angiogenesis [33, 34, 38]. Recent studies revealed CXCL2 is also associated with acquired resistance in breast cancer, colorectal cancer and glioblastoma [30–32]. These phenomena gave us a hint that whether CXCL2 plays an important role in anlotinib resistance. To address this issue, we performed function assays to examine the relevance between CXCL2 and anlotinib resistance. As we expected, our results showed that anlotinib-induced apoptosis and the inhibition of migration and invasion in NCI-H1975 cells were significantly recovered when supplementing exogenous CXCL2, suggesting an important link between CXCL2 and anlotinib resistance.

## Conclusions

In brief, our findings revealed a novel resistant mechanism of anlotinib and provided a basis for circumventing anlotinib resistance.

## Additional files

Additional file 1: This file includes the mRNA levels of anlotinib-responsive 769 genes in NCI-H1975 cells, anlotinib resistant NCI-H1975 cells, and their anlotinib-treated cells. (XLSX 55 kb)

Additional file 2: This file includes the mRNA levels of anlotinib resistance-related 127 genes in NCI-H1975 cells, anlotinib resistant NCI-H1975 cells, and their anlotinib-treated cells. (XLSX 16 kb)

## Abbreviations

CXCL2: C-X-C Motif Chemokine Ligand 2
DAVID: Database for Annotation, Visualization and Integrated Discovery
EGFR: Epidermal Growth Factor Receptor
ENU: N-ethyl-N-nitrosourea
GO: Gene Ontology
KEGG: Kyoto Encyclopedia of Genes and Genomes
NCCN: National Comprehensive Cancer Network
NSCLC: Non-Small Cell Lung Cancer
OS: Overall Survival
PDGFR: Platelet-derived Growth Factor Receptor; FGFR Fibroblast Growth Factor Receptor
PFS: Progression Free Survival
RPKM: Reads per Kilo-base of Transcript per Million
TKI: Tyrosine Kinase Receptor Inhibitor
VEGFR: Vascular Endothelial Growth Factor Receptor 2
