# Correction: Transcriptome profiling analysis reveals that CXCL2 is involved in anlotinib resistance in human lung cancer cells

**DOI:** 10.1186/s12920-022-01309-6

**Published:** 2022-07-08

**Authors:** Jun Lu, Wei Xu, Jie Qian, Shuyuan Wang, Bo Zhang, Lele Zhang, Rong Qiao, Minjuan Hu, Yiming Zhao, Xiaodong Zhao, Baohui Han

**Affiliations:** 1 Department of Pulmonary Medicine, Shanghai Chest Hospital, Shanghai Jiao Tong University, 241 Huaihai West Rd, Shanghai, 200030 China; 2 Shanghai Center for Systems Biomedicine, Shanghai Jiao Tong University, 800 Dong Chuan Rd, Shanghai, 200240 China

## Correction to: BMC Med Genomics 12, 38 (2019) 10.1186/s12920-019-0482-y

Following publication of the original article [1], the authors identified an error in Fig. 4 of their article. The Fig. 4 replacement is published in this correction article.

**Fig. 4:**
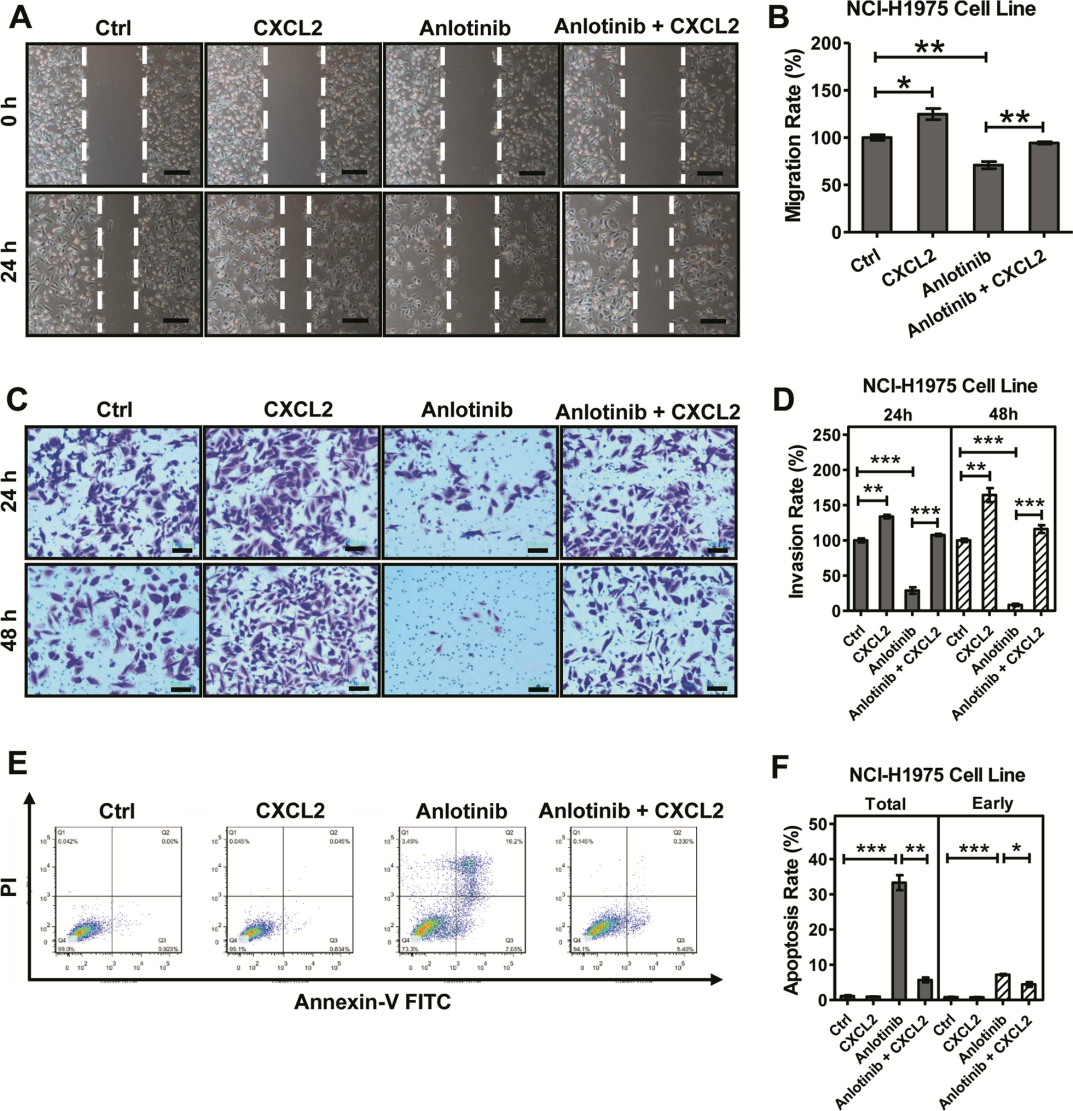
CXCL2 is involved in anlotinib resistance in NCI-H1975 cells. **A**, **B** CXCL2 (50 ng/ml) and anlotinib (4 μg/ml) were performed on NCI-H1975 cells, alone or together for 24 h. Migration rate was examined by wound healing scratch assay. Bars = mean ± SD, *n* = 3, *P < 0.05, **P < 0.01. Scale bar, 100 μm. **C**, **D** CXCL2 (100 ng/ml) and anlotinib (2 μg/ml) were performed on NCI-H1975 cells, alone or together, for 24 h. Invasion rate was analyzed based on transwell assays. Bars = mean ± SD, *n* = 3, **P < 0.01, ***P < 0.001. Scale bar, 100 μm. **E**, **F** NCI-H1975 cells were exposed to CXCL2 (100 ng/ml) and anlotinib (4 μg/ml), alone or together for 24 h. Ratio of total apoptosis and early apoptosis were examined based on flow cytometric detection. Data are shown as mean ± SD, *n* = 3, *P < 0.05, **P < 0.01, ***P < 0.001

## References

[1] Lu J, Xu W, Qian J (2019). Transcriptome profiling analysis reveals that CXCL2 is involved in anlotinib resistance in human lung cancer cells. BMC Med Genomics.

